# Efficient energy management of a low-voltage AC microgrid with renewable and energy storage integration using nonlinear control

**DOI:** 10.1038/s41598-025-22473-6

**Published:** 2025-11-04

**Authors:** Karim El Mezdi, Abdelmounime El Magri, Aziz Watil, Ilyass El Myasse, Nabil El Aadouli, Pankaj Kumar

**Affiliations:** 1 EEIS Laboratory, ENSET Mohammedia, Hassan II University of Casablanca, Mohammedia, 28800 Morocco; 2 LERMA Laboratory, School of Aerospace and Automotive Engineering, International University of Rabat, Rabat, Morocco; 3 LSIB Laboratory, FST Mohammedia, Hassan II University of Casablanca, Mohammedia, 28800 Morocco; 4 Department of Electrical and Electronics Engineering, Manipal Institute of Technology, Manipal Academy of Higher Education, Manipal, Karnataka 576104 India

**Keywords:** Wind turbine, *PV* system, Nonlinear controller, Li-ion battery, *EFM* algorithm, Energy science and technology, Engineering

## Abstract

This paper proposes an enhanced nonlinear control strategy combined with efficient energy flow management for a low-voltage AC microgrid integrating a wind turbine, a photovoltaic system, and a battery energy storage unit. The microgrid operates in a grid-connected configuration, aiming to optimize energy generation, storage, and consumption. To achieve this, a comprehensive mathematical model of the system is developed, and backstepping controllers are designed to fulfill the control objectives. The stability of the closed-loop system is rigorously verified using Lyapunov theory. Furthermore, a novel algorithm is introduced to maximize renewable energy extraction while effectively managing battery storage to enhance system performance and reliability. The proposed approach also ensures grid stability through power factor correction and ensures the load demand is met. Simulation results validate the effectiveness of the control strategy, demonstrating significant improvements in energy efficiency, system stability, and overall dynamic performance under varying load and environmental conditions.

## Introduction

As global energy demand continues to rise, the integration of renewable energy sources (*RES*) into modern power systems has become a vital strategy to ensure sustainability and mitigate environmental concerns^1,2^. Among the various configurations, microgrids represent an innovative solution for decentralized energy management, combining renewable energy generation, energy storage, and load demands in a localized system^3,4^. In particular, the use of photovoltaic (*PV*) systems and wind turbines, coupled with battery energy storage systems (*BESS*), offers a promising approach to achieve energy self-sufficiency and reduce dependency on fossil fuels^5,6^.

Nonlinear control methods are widely used in microgrid management due to their ability to handle the complex and nonlinear dynamics of distributed energy systems^7,8^. Feedback Linearization aims to transform a nonlinear system into a linear one through appropriate state feedback, simplifying the design of linear control techniques^9,10^. While it offers good tracking performance and stability when the system model is well-known, its main drawback is the need for precise system modeling, which can be challenging in practical applications. Additionally, it is sensitive to model errors, especially when system dynamics are uncertain or vary over time^11,12^. Sliding Mode Control (*SMC*) is a robust technique that forces the system to “slide” along a predefined surface, ensuring convergence even in the presence of uncertainties or disturbances^13,14^. While *SMC* excels in robustness, its practical implementation can be hindered by high-frequency oscillations (chattering), which may affect system performance and the longevity of components, such as power converters in a microgrid^15,16^. Moreover, handling these oscillations effectively requires additional design considerations. Fuzzy Logic, on the other hand, is particularly useful in systems with high uncertainty, as it does not rely on precise models but instead uses a set of rules to approximate system behavior^17,18^. Although it is flexible and can deal with uncertainties well, it lacks the optimization rigor needed for ensuring optimal performance in dynamic and complex systems like microgrids^19^. Its rule-based nature may also lead to complexity in rule design and fine-tuning. In contrast, Backstepping offers a systematic approach for controlling nonlinear systems by decomposing the system dynamics into simpler subsystems, ensuring global stability and performance even in the presence of uncertainties and nonlinearities^20–22^. Unlike feedback linearization, it does not require an exact model of the system, making it more robust in practical applications. While *SMC* may provide robustness, the chattering effect is difficult to manage in microgrid applications. Fuzzy logic, although useful for handling uncertainties, lacks the rigor needed for optimal control in dynamic systems. Backstepping, with its ability to ensure stable convergence in multivariable systems, is particularly suited for the integration of renewable energy sources like *PV* and wind turbines, as well as battery storage systems in microgrids. Its robust handling of nonlinearities, coupled with its structured approach, makes it an ideal choice for managing complex and dynamic microgrid environments^23,24^.

Energy flow management (*EFM*) in microgrids has been extensively studied in the literature through a variety of control strategies, as summarized in Table 1. In *DC* microgrids, dynamic optimal power management approaches combining metaheuristic optimization techniques (e.g., *PSO*) with droop-based regulation have been proposed to enhance reliability, but they remain computationally demanding for real-time operation. Other solutions, such as dynamic droop control or filter-based methods applied to hybrid energy storage systems, offer simpler and faster implementations, yet their scalability and robustness remain limited. In *AC* and multi-energy microgrids, optimization-oriented energy management schemes are commonly used, but they mainly operate offline and suffer from high computational complexity, making them less suitable for fast variations. Hybrid *AC*/*DC* microgrids have attracted growing attention in recent years, with research focusing on optimal scheduling as well as *PV*–*BESS* operational strategies under *AC* and *DC* coupled configurations. While scheduling-based methods achieve high efficiency over planning horizons, they lack robustness against rapid fluctuations. Conversely, *PV*–*BESS* coupling approaches provide higher efficiency in energy transfer but face constraints in scalability and integration. Overall, the comparative analysis highlights that existing methods exhibit a trade-off between optimality, computational complexity, and applicability in real-time operation. To address these limitations, the strategy proposed in this work stands out by combining implementation simplicity with efficient and scalable real-time management. It reduces computational complexity while ensuring system stability and reliability, thereby offering clear superiority over existing approaches.

**Table 1:** Comparative table of microgrid control strategies.

**Refs**	**Microgrid type**	**Control strategy**	**EFM**	**Complexity (Online/Offline)**	**Main idea**	**Remark**
^25–27^	*DC*	Dynamic optimal power management (PSO + droop)	Yes	Hybrid (Offline–PSO/Online–droop) – Medium	Reliable operation of islanded *DC* microgrids with *PV*, battery and generator using *PSO* scheduling and droop	Balances reliability and economics; *PSO* is computationally heavy, droop stabilizes *DC* bus
^28–31^	*DC*	Dynamic droop control	Yes	Online – Low	Variable droop resistance for DC bus voltage regulation and proportional current sharing	Improves voltage profile but requires frequent recalculations; limited scalability
^32–35^	*DC*	Adaptive Filter-Based Method (*FBM*)	Yes	Online – Medium	Hybrid battery + supercapacitor with adaptive SOC/capacity-based control	Enhances dynamics and voltage stability; validated in simulation
^36,37^	*AC*/Multi-energy	Voltage-oriented EMS (Optimization)	Yes	Offline – High	Multi-energy *EMS* for residential *AC* microgrids (electric + thermal) with voltage-oriented optimization	Effective for planning; not flexible in real time
^38,39^	Hybrid ($AC+DC$)	Optimal scheduling & coordinated control	Yes	Offline – High	Optimization-based scheduling for coordinated *AC*/*DC* resource and storage management	High efficiency in planning; less robust to fast fluctuations
^40–42^	Hybrid ($AC+DC$)	*PV*-*BESS* operational strategies (*AC*/*DC* coupling)	Yes	Online – Low/Medium	Comparative analysis of *AC* coupled vs *DC* coupled *PV*-*BESS* systems	*DC*-coupled offers higher efficiency but limited scalability

**Fig. 1:**
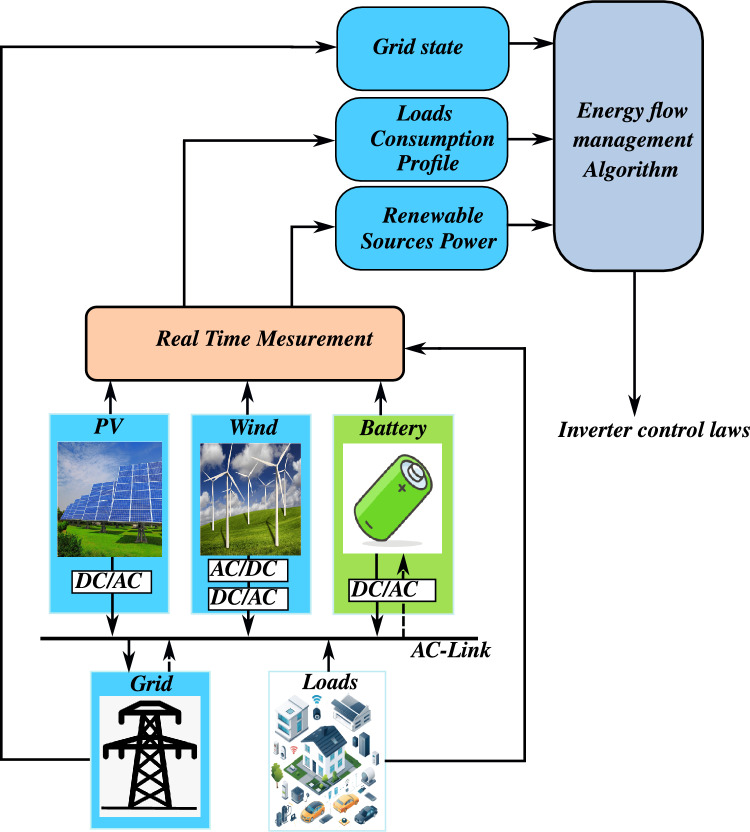
The proposed grid-connected low-voltage *AC* microgrid with renewable integration and energy storage.

This paper focuses on the development of a nonlinear control framework enhanced by a new energy flow management algorithm for a low voltage *AC* microgrid integrating a wind turbine, a photovoltaic (*PV*) system, and a battery energy storage system (*BESS*), as illustrated in Fig. 1. The proposed approach aims to achieve two main objectives: (1) maximize power extraction from renewable energy sources and (2) ensure effective power factor correction (*PFC*) at the grid interface. The microgrid topology includes a single-phase grid connection through power electronic converters, which play a critical role in implementing the control strategy.

The novelty of this work lies in the synergistic application of nonlinear control techniques and a new energy flow management algorithm. By combining the strengths of these methods, the proposed system can handle the uncertainties and nonlinearities inherent in renewable energy generation. These uncertainties include the stochastic variability of solar irradiance, wind speed, and load demand, as well as nonlinear effects in converters and storage systems. The nonlinear control and energy flow management strategy dynamically mitigates these effects, ensuring stable and reliable operation under diverse conditions. Moreover, this control framework ensures balanced power sharing among the components, optimal utilization of the energy storage system, and improved power quality at the grid connection point.

Through detailed simulations and performance evaluations, this study demonstrates the effectiveness of the nonlinear control approach enhanced by *EFM* algorithm. The results highlight its ability to achieve the desired objectives under dynamic operating conditions, making it a viable solution for modern energy systems.

**Fig. 2:**
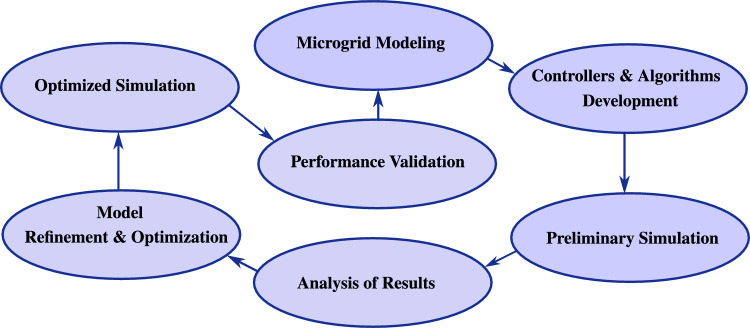
Simulation-driven validation methodology for the proposed *AC* microgrid.

Figure 2 outlines the methodology employed in this study. The process commences with the modeling of the microgrid, incorporating multiple energy sources, storage systems, and load components. Subsequently, nonlinear controllers and algorithms are designed to facilitate energy management and the optimization of power flows. An initial simulation is conducted to evaluate the dynamic behavior of the modeled system under the proposed control strategies. The simulation outcomes are then subjected to detailed analysis in order to assess system performance and identify potential limitations. Based on these findings, refinements are introduced to the model, followed by an optimized simulation to validate the achieved improvements and to confirm the stability of the microgrid. The methodology is finalized through the validation of the developed controllers and algorithms against predefined performance benchmarks.

An additional step of reliability quantification is included, combining stability analysis with KPI-based performance evaluation^3,4^.

## System modelling

**Fig. 3:**
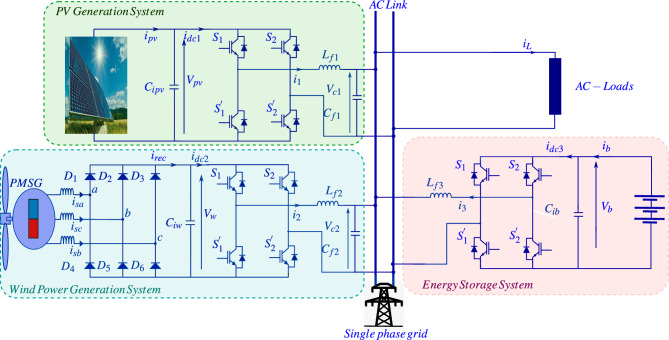
Schematic diagram of the proposed low voltage *AC* microgrid.

The proposed model for an energy conversion system, as shown in Fig. 3, has been integrated with the *PV* panel, a wind turbine, and a battery storage system to connect with the single-phase *AC* grid. The photovoltaic (*PV*) panel is directly connected to the grid with an inverter. The variable *AC* output of the wind turbine has been connected through a diode rectifier followed by an inverter to make it compatible with the grid. The interfacing of the battery via its inverter acts like an energy buffer, which is supposed to supply surplus energy for extra power. This configuration must ensure that energy supply to either the grid or to the connected loads is continuous and effective.

### PViGeneration system modelling

The configuration of a photovoltaic production system, which consists of a *PV* array, a capacitor $C_{ipv}$, and an $L-C$ filter, connects the single-phase bridge inverter composed of four IGBTs with anti-parallel diodes to the power supply network. This subsystem is represented by the following system of differential equations obtained from the application of Kirchhoff’s electrical laws. The instantaneous model derived above truly represents the dynamics of the *PV* inverter. However, due to the switched nature of the control input $\mu _{pv}$, this model is not suitable for control design. Most nonlinear control strategies are designed for systems with continuous control inputs. Thus, an averaged version of the inverter model will be used for the control of the *PV* system:

1a $$\begin{aligned} \dot{x}_{11}&= \frac{x_{12}}{L_{g}}-\frac{v_{g}}{L_{g}}\end{aligned}$$

1b $$\begin{aligned} \dot{x}_{12}&= \frac{1}{C_{f1}} x_{13}-\frac{1}{C_{f1}} x_{11}\end{aligned}$$

1c $$\begin{aligned} \dot{x}_{13}&=-\frac{x_{12}}{L_{f1}}+\mu _{pv} \frac{V_{pv}}{L_{f1}}\end{aligned}$$

1d $$\begin{aligned} \dot{x}_{14}&= \frac{2}{C_{ipv}} V_{pv} i_{p v}-\frac{2}{C_{ipv}} i_{dc1} V_{pv} \end{aligned}$$

Where $x_{11}=\bar{i}_{g}$ is the line current in inductor $L_g$, $x_{12}=\bar{V}_{c1}$ is the voltage across capacitor $C_{f1}$, $x_{13}=\bar{i}_{1}$ is the inverter output current, $x_{14}=\bar{V}^2_{pv}$ denotes the voltage across capacitor $C_{ipv}$ (*PV* voltage), $\mu _{pv}$ is the switching function that accepts values from the discrete set $\{-1,1\}$, $V_g = \sqrt{2}E \cos (\omega _{g} t)$ is the sinusoidal grid voltage (with known constants *E*, $\omega _g$), $i_{pv}$ is the *PV* output current, $i_{dc1}$ is the inverter input current, and $L_{f1}$ is the filter inductance.

### Wind power generation system modelling

The wind energy conversion system consists of a wind turbine, an input capacitor $C_{iw}$, a diode rectifier and an $L-C$ filter that connects the single-phase bridge inverter, which is composed of four IGBTs with anti-parallel diodes, to the power supply network. This subsystem is described by the following set of differential equations derived using Kirchhoff’s laws. For the wind power generation system, the instantaneous model accurately reflects the behavior of the inverter. Nevertheless, the switched control input $\mu _{w}$, poses challenges for applying nonlinear control methods, which are generally designed for continuous control inputs. To address this limitation, the averaged model will be employed for control purposes:

2a $$\begin{aligned} \dot{x}_{21}&= \frac{x_{22}}{L_{g}}-\frac{v_{g}}{L_{g}}\end{aligned}$$

2b $$\begin{aligned} \dot{x}_{22}&= \frac{1}{C_{f2}} x_{23}-\frac{1}{C_{f2}} x_{21}\end{aligned}$$

2c $$\begin{aligned} \dot{x}_{23}&=-\frac{x_{22}}{L_{f2}}+\mu _{w} \frac{V_{w}}{L_{f2}}\end{aligned}$$

2d $$\begin{aligned} \dot{x}_{24}&= \frac{2}{C_{iw}} V_{w} i_{w}-\frac{2}{C_{iw}} i_{dc2} V_{w} \end{aligned}$$

Where $x_{21}=\bar{i}_{g}$ is the line current in inductor $L_g$, $x_{22}=\bar{V}_{c2}$ is the voltage across capacitor $C_{f2}$, $x_{23}=\bar{i}_{2}$ is the inverter output current, $x_{24}=\bar{V}^2_{w}$ denotes the capacitor $C_{iw}$ voltage (Wind turbine voltage), $\mu _{w}$ is the switching function that accepts values from the discrete set $\{-1,1\}$, $V_g = \sqrt{2}E \cos (\omega _{g} t)$ is the sinusoidal grid voltage (with known constants *E*, $\omega _g$), $i_{rec}$ designates the diode rectifier output current, $i_{dc2}$ is the inverter input current, and $L_{f2}$ is the filter inductance.

### Energy storage system modelling

**Fig. 4:**
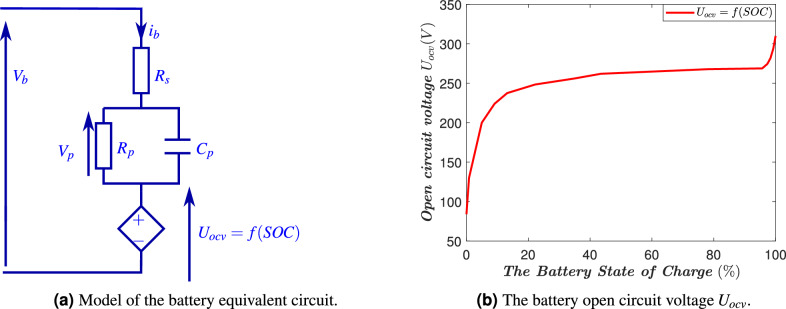
Equivalent electrical circuit of Lithium-ion battery.

The energy storage system configuration is constituted by a battery, an input capacitor $C_{ib}$, an inductor $L_{f3}$, and an inverter that connects the power supply network to the single-phase bridge switching composed of four IGBTs with anti-parallel diodes. In this work, an electrical model for the battery is adopted; indeed, every real battery is a kind of parallel *RC* circuit in series with the internal resistance of the battery. This consists of an equivalent circuit with $R_s$ accounting for the ohmic losses of the battery and a first-order $R_pC_p$ network that models the long-term transient polarization effect, as shown in Fig. 4a. The current through the circuit is the battery current, $i_b$, and the open-circuit voltage, $U_{ocv}$, a nonlinear function of the state of charge (*SOC*), expressed as $U_{ocv} = f(SOC)$, as shown in Fig. 4b. This subsystem is modeled using Kirchhoff’s laws.

The instantaneous model accurately represents the operation of the energy storage inverter. However, for control design, an averaged version of the model will be adopted:

3a $$\begin{aligned} \dot{x}_{31}&=-\frac{V_{g}}{L_{f3}}+\mu _{b} \frac{\sqrt{x_{32}}}{L_{f3}}\end{aligned}$$

3b $$\begin{aligned} \dot{x}_{32}&= \frac{2}{C_{ib}}i_{b}\sqrt{x_{32}}-\frac{2}{C_{ib}} i_{dc3}\sqrt{x_{32}}\end{aligned}$$

3c $$\begin{aligned} \dot{x}_{33}&= \frac{i_b}{C_{p}}-\frac{x_{33}}{C_{p} R_{p}} \end{aligned}$$

Where $x_{31}=\bar{i}_{3}$ is the inverter output current, $x_{32}=\bar{V}^2_{b}$ denotes the battery voltage, $x_{33}=\bar{V}_{p}$ is the polarization voltage of the capacitor $C_{p}$, $\mu _{b}$ is the switching function that accepts values from the discrete set $\{-1,1\}$, $V_g = \sqrt{2}E \cos (\omega _{g} t)$ is the sinusoidal grid voltage (with known constants *E*, $\omega _g$) and $i_{dc3}$ is the inverter input current.

#### Remark 1

: Parameter Selection Criteria

The parameters of the photovoltaic (*PV*), wind turbine (*WT*), and battery storage systems were selected using a resource-based and standards-driven methodology, consistent with^43^.

For the *PV* subsystem, the nominal capacity was determined as

$$P_{PV} = \eta _{PV} \times G \times A,$$

where *G* is the average solar irradiation, *A* the module surface, and $\eta _{PV}$ the efficiency corrected by the temperature coefficient. A derating factor and performance ratio were included. The PV module selection complies with IEC 61215 and IEC 61730 standards.

For the *WT* subsystem, turbines were chosen according to the Weibull-distributed wind speed profile of the site. The expected energy yield was obtained by integrating the turbine power curve *P*(*V*) over the wind speed probability density function. The selection process follows IEC 61400 standards for wind turbine performance.

For the battery subsystem, the required capacity was computed as

$$C_{bat} = \frac{E_{deficit} \times \text {Autonomy}}{\eta _{bat} \times DoD},$$

considering State of Charge (*SOC*) limits, Depth of Discharge (*DoD*), and round-trip efficiency. The sizing follows IEC 62619 guidelines.

## Nonlinear controller design

### Control objectives

The proposed control framework is developed to achieve four key objectives that ensure the efficient and stable operation of the microgrid. These objectives focus on optimizing power extraction from renewable energy sources, regulating the battery’s charging and discharging processes, and maintaining grid stability with high power quality:

(i) ***Maximum/Adaptive Power Point Tracking (MPPT/APPT):*** The voltages of the photovoltaic system ($V_{pv}$) and the wind turbine ($V_{w}$) must accurately follow their respective optimal reference values ($V_{pvopt}$ and $V_{wopt}$). These reference values are dynamically determined by two optimizers, thereby ensuring that both systems continuously operate at their optimal power point.
(ii) ***Power Factor Correction (PFC):*** The grid current ($i_g$) must be sinusoidal, matching the frequency and phase of the grid voltage ($V_g$). The last two control objectives depend on the battery’s state of charge (*SOC*):
(iii) ***Constant Current (CC) Mode:*** During this stage, the battery current $i_b$ is regulated to follow a constant reference value $i^*_{b}$, which represents the maximum allowable charging current. This mode continues until the battery voltage reaches its maximum charging threshold, typically corresponding to about $70{-}80\%$ of the battery *SOC*.
(iv) ***Constant Voltage (CV) Mode:*** Once the maximum battery voltage is reached, the control switches to this mode. At this stage, the voltage is maintained constant at a fixed reference value $V^*_{b}$, while the charging current gradually decreases. When the battery *SOC* reaches 100%, the charging current becomes nearly zero.

### PV inverter controller design

The current $x_{11}$ is required to track a reference $x_{11}^{*}$ that is sinusoidaliand proportionalito theivoltage $V_g$, expressed as:

4 $$\begin{aligned} x_{11}^{*} = k_{1}V_g \end{aligned}$$

To achieve this goal, a control law is derived usingithe backsteppingiapproach, inspired by the methods presented in^44,45^. The backstepping controller is implemented in three steps.

***Step 1:*** Define the tracking error as $e_{11} = x_{11} - x_{11}^{*}$, using equation (1a),

the error dynamics can be expressed as:

5 $$\begin{aligned} \dot{e}_{11} = \frac{x_{12}}{L_g} - \frac{V_g}{L_g} - \dot{x}_{11}^* \end{aligned}$$

To stabilize the system,iconsider theiquadratic Lyapunovifunction:

6 $$\begin{aligned} V_{11} = 0.5e_{11}^2 \end{aligned}$$

The time derivative $\dot{V}_{11}$ becomes negative definite if the virtual input $x_{12}$ is chosen as follows:

7 $$\begin{aligned} x_{12}^* = L_g(-c_{11}e_{11} + \frac{V_g}{L_g} + \dot{x}_{11}^{*}) \end{aligned}$$

where $c_{11}$ is a positive design parameter. When $x_{12}$ converges to $x_{12}^*$, the error $e_{11}$ evolves as $\dot{e}_{11} = -c_{11}e_{11}$.

***Step 2:*** Define the tracking error for $x_{12}$ as $e_{12} = x_{12} - x_{12}^{*}$, using equation (1b), The corresponding dynamics is given by:

8 $$\begin{aligned} \dot{e}_{12} = -\frac{1}{C_{f1}}x_{13} - \frac{1}{C_{f1}}x_{11} - \dot{x}_{12}^* \end{aligned}$$

Using the Lyapunov function:

9 $$\begin{aligned} V_{12} = 0.5e_{12}^2 \end{aligned}$$

and selecting the virtual input $x_{13}$ as:

10 $$\begin{aligned} x_{13}^* = C_{f1}(c_{12}e_{12} - \frac{1}{C_{f1}}x_{11} - \dot{x}_{12}^*) \end{aligned}$$

where $c_{12}$ is a positive design parameter, $\dot{V}_2$ becomes negative definite, ensuring system stability.

***Step 3:*** Similarly, define the tracking error for $x_{13}$ as:

11 $$\begin{aligned} e_{13} = x_{13} - x_{13}^* \end{aligned}$$

A Lyapunov function $V_{13} = 0.5e_{13}^2$ is chosen, and the stabilizing control law is defined as:

12 $$\begin{aligned} \mu _{pv} = \frac{L_{f1}}{V_{pv}}(-c_{13}e_{13} + \frac{x_{12}}{L_{f1}} + \dot{x}_3^*) \end{aligned}$$

where $c_{13}$ is a positive parameter, guaranteeing global stability of the system.

For the *PV* voltage regulation, the tracking error is defined as:

13 $$\begin{aligned} e_{14} = x_{14} - x_{14}^* \end{aligned}$$

Using a Lyapunov function $V_{14} = 0.5e_{14}^2$, the stabilizing control law for the ratio $k_1$ is given by:

14 $$\begin{aligned} k_1 = \frac{1}{V_g^2}\Big [\frac{C_{ipv}}{2}(c_{14}e_{14} - \dot{x}_{14}^*) + \sqrt{x_{14}}i{pv}\Big ] \end{aligned}$$

where $c_{14}$ is a positive design parameter.

**Fig. 5:**
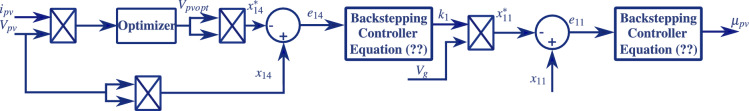
Control block diagram of the *PV* generation system.

To clarify the synthesis of the backstepping controller for the *PV* generation system, Fig. 5 depicts the proposed control strategy, emphasizing the interactions between the reference signals, the power extraction optimizer, and the regulation loops. By associating the mathematical equations with the functional control diagram of the *PV* system, the role of each variable is explicitly identified, thereby enhancing the readability of the control design and facilitating the understanding of the proposed methodology.

#### Proposition 1

Consider the *PV* generation system modeled by the state-space equations (1a)-(1d) with the control input defined in (12), where $c_{11}$, $c_{12}$, and $c_{13}$ are positiveidesign parameters. If the reference signal $x_{11}^{*} = kV{g}$iand its derivative exist,ithe system exhibits the followingiproperties:

1. Theiclosed-loopidynamics of the variables ($e_{11}$, $e_{12}$, $e_{13}$) are governed by:
  15 $$\begin{aligned} \begin{aligned} \dot{e}_{11}&= -c_{11}e_{11} + e_{12}, \\ \dot{e}_{12}&= -c_{12}e_{12} - e_{11}, \\ \dot{e}_{13}&= -c_{13}e_{13}. \end{aligned} \end{aligned}$$
  This linear closed-loopisystem (15) is globallyiasymptoticallyistable under any initialiconditions. As a result, the *PFC* objective is asymptotically satisfied on average.
2. Moreover, if $k_1$iconvergesito a finite value,ithe trackingierror of the *PV* voltage, $\dot{e}_{14} = \dot{x}_{14} - \dot{x}_{14}^*$, follows the differential equation $\dot{e}_{14} = -c_{14}e_{14}$, where $c_{14}$ is aipositiveiconstant. Consequently, the closed-loop system achieves global exponential stability, ensuring that the error $e_{14}$ decays exponentially regardless of the initial conditions. This guarantees that the *PV* voltage accurately tracksithe referenceivoltage providedibyioptimizer.

### Wind power inverter controller design

The current $x_{21}$ is required to track a reference $x_{21}^{*}$ that is sinusoidal and proportional to the voltage $V_g$, expressed as:

16 $$\begin{aligned} x_{21}^{*} = k_2V_g \end{aligned}$$

To achieve this objective, in the same way as before and by the same method. The backstepping controller is implemented in three steps.

***Step 1:*** Define the tracking error as:

17 $$\begin{aligned} e_{21} = x_{21} - x_{21}^{*} \end{aligned}$$

From equation (17), the error dynamics can be expressed as:

18 $$\begin{aligned} \dot{e}_{21} = \frac{x_{22}}{L_g} - \frac{V_g}{L_g} - \dot{x}_{21}^* \end{aligned}$$

To stabilize the system, consider the quadratic Lyapunov function:

19 $$\begin{aligned} V_{21} = 0.5e_{21}^2 \end{aligned}$$

The time derivative $\dot{V}_{21}$ becomes negative definite if the virtual input $x_{22}$ is chosen as follows:

20 $$\begin{aligned} x_{22}^* = L_g(-c_{21}e_{21} + \frac{V_g}{L_g} + \dot{x}_{21}^{*}) \end{aligned}$$

where $c_{22}$ is a positive design parameter. When $x_{22}$ converges to $x_{22}^*$, the error $e_{21}$ evolves as $\dot{e}_{21} = -c_{11}e_{21}$.

***Step 2:*** Define the tracking error for $x_{22}$:

21 $$\begin{aligned} e_{22} = x_{22} - x_{22}^{*} \end{aligned}$$

The corresponding dynamics is given by:

22 $$\begin{aligned} \dot{e}_{22} = -\frac{1}{C_{f2}}x_{23} - \frac{1}{C_{f2}}x_{21} - \dot{x}_{22}^* \end{aligned}$$

Using the Lyapunov function:

23 $$\begin{aligned} V_{22} = 0.5e_{22}^2 \end{aligned}$$

and selecting the virtual input $x_{23}$ as:

24 $$\begin{aligned} x_{23}^* = C_{f2}(c_{22}e_{22} - \frac{1}{C_{f2}}x_{21} - \dot{x}_{22}^*) \end{aligned}$$

where $c_{22}$ is a positive design parameter, $\dot{V}_2$ becomes negative definite, ensuring system stability.

***Step 3:*** Similarly, define the tracking error for $x_{23}$ as:

25 $$\begin{aligned} e_{23} = x_{23} - x_{23}^* \end{aligned}$$

A Lyapunov function $V_{23} = 0.5e_{23}^2$ is chosen, and the stabilizing control law is defined as:

26 $$\begin{aligned} \mu _{w} = \frac{L_{f2}}{V_{w}}(-c_{23}e_{23} + \frac{x_{22}}{L_{f2}} + \dot{x}_{23}^*) \end{aligned}$$

where $c_{23}$ is a positive parameter, guaranteeing global stability of the system.

For the wind turbine voltage, the tracking error is defined as:

27 $$\begin{aligned} e_{24} = x_{24} - x_{24}^* \end{aligned}$$

Using a Lyapunov function $V_{24} = 0.5e_{24}^2$, the stabilizing control law for the ratio $k_2$ is given by:

28 $$\begin{aligned} k_2 = \frac{1}{V_g^2}\Big [\frac{C_{iw}}{2}(c_{24}e_{24} - \dot{x}_{24}^*) + \sqrt{x_{24}}i_{rec}\Big ] \end{aligned}$$

where $c_{24}$ is a positive design parameter.

**Fig. 6:**
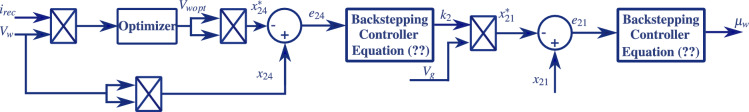
Control block diagram of the wind power generation system.

Figure 6 presents the control block diagram illustrating the strategy adopted for the wind energy generation system. This graphical representation complements the mathematical formulation by clarifying the role of the control variables and showing the coordination of the regulation loops, thereby facilitating the understanding of the operation of this subsystem within the overall microgrid.

#### Proposition 2

Consider the wind power generation system modeled by the state-space equations (2a)-(2d) with the control input defined in (26), where $c_{21}$, $c_{22}$, and $c_{23}$ are positive designiparameters.iIf theireference signal $x_{21}^{*} = kV{g}$iand itsiderivative exist,ithe system exhibits the followingiproperties:

1. Theiclosed-loop dynamics of the variables ($e_{21}$, $e_{22}$, $e_{23}$) are governed by:
  29 $$\begin{aligned} \begin{aligned} \dot{e}_{21}&= -c_{21}e_{21} + e_{22}, \\ \dot{e}_{22}&= -c_{22}e_{22} - e_{21}, \\ \dot{e}_{23}&= -c_{23}e_{23}. \end{aligned} \end{aligned}$$
  This linear closed-loopisystem (29) is globallyiasymptoticallyistable under any initialiconditions. As a result, the *PFC* objective is asymptotically satisfied on average.
2. Moreover, if $k_2$ convergesito aifinite value, the trackingierror of the wind turbine voltage, $\dot{e}_{24} = \dot{x}_{24} - \dot{x}_{24}^*$, follows the differential equation $\dot{e}_{24} = -c_{24}e_{24}$, where $c_{24}$ is a positive constant. Consequently, the closed-loop system achieves global exponential stability, ensuring that the error $e_{24}$ decays exponentially regardlessiof the initialiconditions. This guarantees that the wind turbine voltage accurately tracks theireference voltageiprovided by ioptimizer.

### Energy Storage inverter controller design

The current $x_{31}$ is required to track a reference $x_{31}^{*}$ that is sinusoidal and proportional to the voltage $V_g$, expressed as:

30 $$\begin{aligned} x_{31}^{*} = k_3V_g \end{aligned}$$

To achieve this goal, define the tracking error as:

31 $$\begin{aligned} e_{31} = x_{31} - x_{31}^{*} \end{aligned}$$

From equation (31), the error dynamics can be expressed as:

32 $$\begin{aligned} \dot{e}_{31} =- \frac{V_{g}}{L_{f3}} - \mu _{b}\frac{\sqrt{x_{32}}}{L_{f3}} - \dot{x}_{31}^* \end{aligned}$$

A Lyapunov function $V_1 = 0.5e_{31}^2$ is chosen, and the stabilizing control law is defined as:

33 $$\begin{aligned} \mu _{b} = \frac{L_{f3}}{\sqrt{x_{32}}}(-c_{31}e_{31} + \frac{V_{g}}{L_{f3}} + \dot{x}_{31}^*) \end{aligned}$$

where $c_{31}$ is a positive parameter, guaranteeing global stability of the system.

The control design of the inverter will be conducted to ensure the charging and discharging of the battery. These two modes are known as *CC* mode and *CV* mode.

#### CC mode controller design

Recall that the control objective in *CC* mode is to enforce the battery current $i_{b}$ to track its desired constant value $i^*_{b}$. Using the backstepping design technique, it follows from the subsystem (3a-3c). Let first consider the following assumption:

##### Assumption 1

Knowing that the battery open circuit voltage $U_{ocv}$ has a slow change rate compared with the battery current dynamics. So one can assume that $\dot{U}_{ocv}\approx 0$. Let us introduce the battery current tracking error:

34 $$\begin{aligned} e_{32}=i_{b}-i_{b}^*=\frac{x_{32}-x_{33}-U_{ocv}}{R_{s}}-i_{b}^* \end{aligned}$$

In view of equation (34), the dynamic of the above error undergo the following differential equation:

$$\dot{e}_{32}=\frac{\dot{x}_{32}-\dot{x}_{33}}{R_{s}}=-\frac{\dot{x}_{33}}{R_{s}}+\frac{\dot{x}_{32}^2}{2{x}_{32}R_{s}}$$

wich gives:

35 $$\begin{aligned} \dot{e}_{32}=-\frac{\dot{x}_{33}}{R_{s}}+\frac{k_{3cc}V_g^2-i_{b}\sqrt{x_{32}}}{R_{s}C_{ib}\sqrt{x_{32}}} \end{aligned}$$

Using a Lyapunov function $V_2 = 0.5e_{32}^2$, the stabilizing control law for the ratio $k_{3cc}$ is given by:

36 $$\begin{aligned} k_{3cc} = \frac{1}{V_g^2}\Big [R_{s}C_{ib}\sqrt{x_{32}}[-c_{32}e_{32}+\frac{\dot{x}_{33}}{R_{s}}]+i_{b}\sqrt{x_{32}}\Big ] \end{aligned}$$

where $c_{32}$ is a positive design parameter.

#### CV mode controller design

In this subsection, the control objective is to maintain the battery voltage $V_{b}$ and equal to its constant reference $V_{b}^*$ during the *CV* mode. Again, using the backstepping tecchnique, its follows from the subsystem (3a-3c), let us introduce the voltage tracking error as follows:

37 $$\begin{aligned} e_{33}=x_{32}-V_{b}^{2*} \end{aligned}$$

Considering that $\dot{V}_{b}^{2*}=0$ and using equation (3b), the time derivation of equation (37) yields:

38 $$\begin{aligned} \dot{e}_{33}=\dot{x}_{32}=\frac{2}{C_{ib}}[k_{3cv}V_g^2-i_b\sqrt{x_{32}}] \end{aligned}$$

Using a Lyapunov function $V_3 = 0.5e_{33}^2$, the stabilizing control law for the ratio $k_{3cv}$ is given by:

39 $$\begin{aligned} k_{3cv} = \frac{1}{V_g^2}\Big [-\frac{C_{ib}}{2}c_{33}e_{33}+i_b\sqrt{x_{32}}\Big ] \end{aligned}$$

**Fig. 7:**
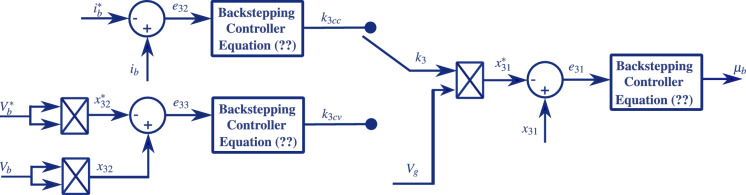
Control block diagram of the *BESS*.

The control block diagram of the *BESS*, shown in Fig. 7, illustrates the adopted strategy, with particular emphasis on the selection between the *CC* and *CV* charging modes according to the battery *SOC*. This representation complements the mathematical formulation by explicitly defining the role of each control variable and clarifying the interaction between the regulation loops, thereby enhancing the understanding of the *BESS* charging and discharging processes as well as its contribution to the overall operation of the microgrid.

## Energy flow management

Energy flow management (*EFM*) in a low voltage *AC* microgrid, incorporating renewable sources such as photovoltaic and wind energy, along with a battery storage system and alternative loads, is essential for ensuring the network’s stability and efficiency. It optimizes the use of available resources, reduces energy losses, and ensures a reliable power supply for connected loads. By balancing energy production and consumption, this management also promotes system sustainability and facilitates the seamless integration of renewable energy into the electrical grid.

**Fig. 8:**
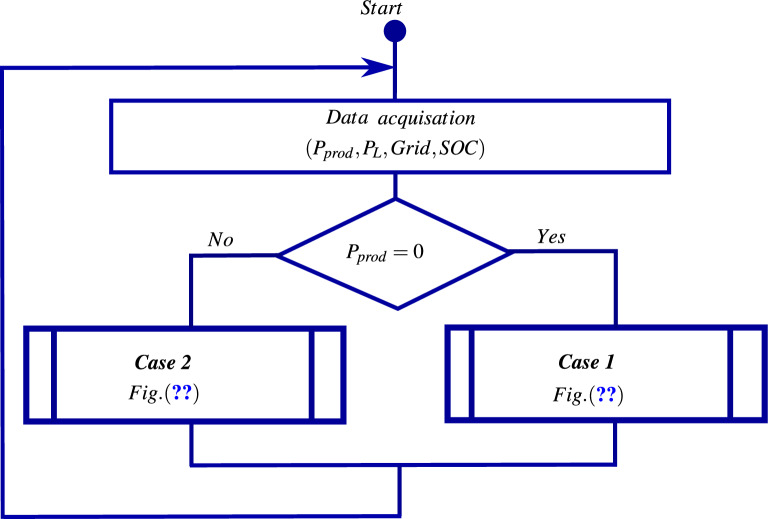
The energy flow management flowchart.

**Fig. 9:**
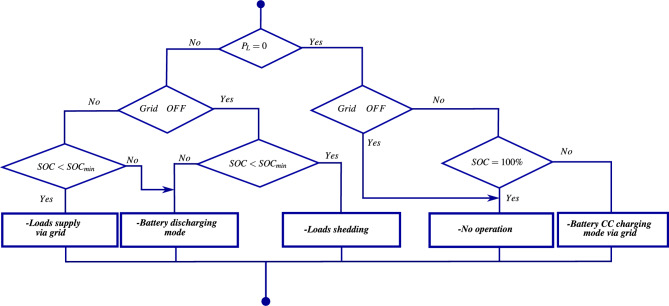
The energy flow management flowchart (Case 1).

**Fig. 10:**
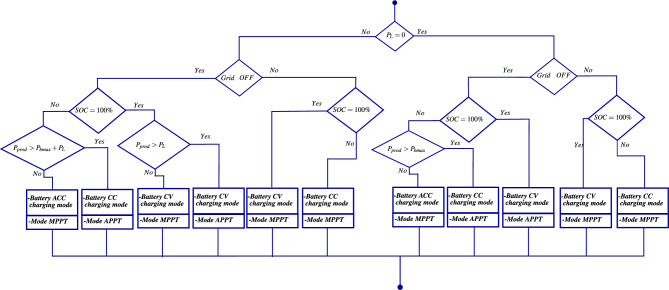
The energy flow management flowchart (Case 2).

Taking into account the power provided by renewable energy sources, the battery state of charge, the energy demand of *AC* loads, and the availability of the grid, several energy flow management scenarios are considered to balance the power exchange between the loads and various energy sources. These scenarios aim to minimize system costs (economic aspect), ensure grid stability, and improve power quality (technical aspect). To meet these *EFM* requirements, a flowchart is proposed in Figs. 8, 9 and 10 to illustrate the following operating modes.

## Simulation results

**Fig. 11:**
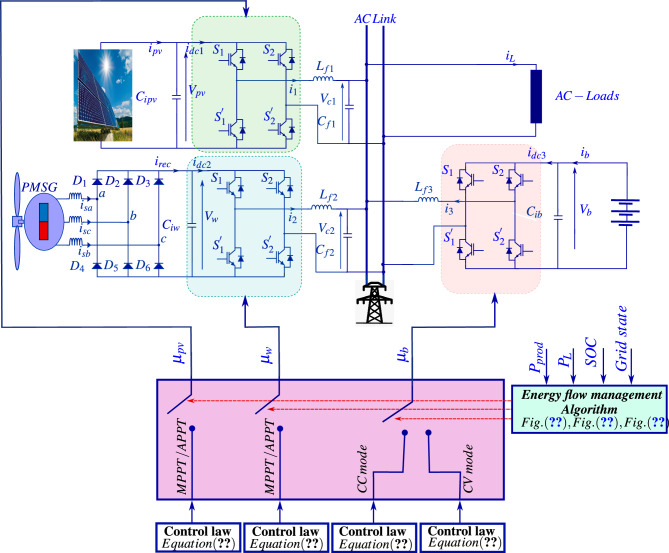
Block diagram of the proposed controller implementation.

The experimental setup described by Fig. 11 has been simulated, within the Matlab/Simulink/SimPowerSystems environment. The system characteristics and the design parameters of the controllers are summarized in Tables 2 and 3.

**Table 2:** system characteristics.

Characteristics	Values	Characteristics	Values
**PV array**		***Wind turbine***	
Parallel strings	1	Nominal power	$P_t=9\,KW$
Series-connected modules per string	20	Turbine radius	$R_t=0.9\,m$
Cells per module $N_{cell}$	60	Blade pitch	$\beta =2\,^\circ$
Maximum power $P_{m}(W)$	320	***Aero-generator***	
Short circuit current $I_{S C }(A)$	9.1	Nominal power	$P_n=9\,KW$
Open circuit voltage $V_{o c}(V)$	41.2	Number of pole pairs	$p=2$
Voltage at max power point $V_{m p}(V)$	35.3	Nominal speed	$\Omega _n=157\,rd/s$
Current at max power point $I_{m p}(A)$	9.0	Stator resistor	$R_s=0.6\,\Omega$
Temperature coefficient of $I_{s c}(\%/deg.C)$	0.05	Stator cyclic inductor	$L_s=0.0094\,H$
Temperature coefficient of $V_{o c}(\%/deg.C)$	−0.36	Rotor flux	$\phi _r=0.8\,Wb$
**Single phase supply network**	220*V*/50*Hz*	Total inertia	$J=0.01\,Nm/rd/s^2$
Capacitor	$C_{ipv} = 10 \, mF$	Total viscous friction	$f=0.01 \,Kg.m^2.s^{-1}$
Capacitor	$C_{iw} = 1 \, mF$	**Li-ion battery**	
Capacitor	$C_{ib} = 30 \, mF$	Internal resistor	$R_{s} = 100 \, m\Omega$
Inductor	$L_{f1} = 20\, \mu H$	Polarization resistor	$R_{p} = 1 \, \mu \Omega$
Inductor	$L_{f2} = 20\, \mu H$	Polarization capacitor	$C_{p} = 5000 \, F$
Inductor	$L_{f3} = 20\, \mu H$	Nominal voltage	$309.6\, V$
**DC/AC inverter**		Maximal voltage	$312\, V$
Resistor	$R_{g} = 10 \, m\Omega$	Nominal capacity	$Q_{n} = 500 \, Ah$
Inductor	$L_{g} = 10\, mH$	Capacitor	$C_{f1} = 20 \, nF$
Modulation frequency	$F_{m}=25KHz$	Capacitor	$C_{f2} = 20 \, nF$

**Table 3:** Controller design parameters.

Controller parameters	$c_{11}=2000$ ;$c_{12}=10$ ;$c_{13}=2000$ ;$c_{14}=10$ ;
	$c_{21}=10000$ ;$c_{22}=1000$ ;$c_{23}=8000$ ;$c_{24}=50$ ;
	$c_{31}=5000$ ;$c_{32}=1000$ ;$c_{33}=1000$ .

### Case with energy generation

**Fig. 12:**
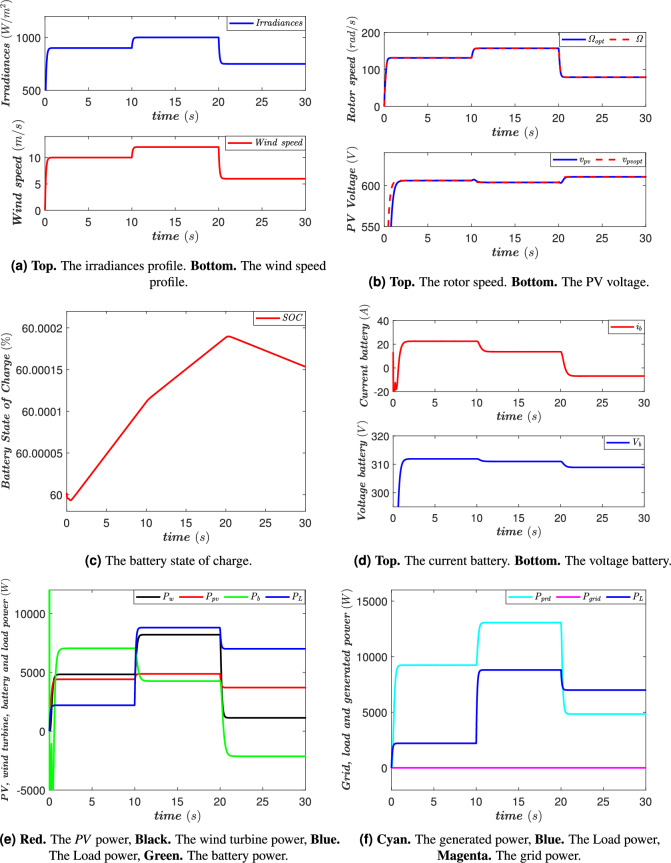
The power flow management system’s performance in the case with energy generation.

In this case, energy production is ensured by the *PV* and the wind turbine, with the irradiance and wind speed profiles illustrated in Fig. 12a. These environmental conditions directly influence the amount of energy produced by these renewable sources and, consequently, the energy management within the system. During the time interval [0, 10 s], the battery charges using the excess energy supplied by the renewable resources (Fig. 12c). In this phase, the power generated by the *PV* and the wind turbine is sufficient not only to supply the *AC* loads but also to charge the battery. It is important to note that, during this period, the grid does not contribute to supplying the loads or charging the battery, meaning that the entire system operates in full autonomy using renewable energy sources (Fig. 12f). Between [10, 20 s], the load consumption gradually increases, as illustrated in Fig. 12e. Despite this rise in demand, renewable energy production remains sufficient to cover the power requirements of the *AC* loads, and the battery continues to charge. However, the charging current dynamics evolve based on the availability of excess energy, as shown in Fig. 12c and d. This phase highlights the system’s adaptability to a moderate increase in demand without requiring additional energy from the grid. Then, during the interval [20, 30 s], a significant shift in the system’s energy balance is observed. The power demand of the *AC* loads now exceeds the power generated by the *PV* and the wind turbine (Fig. 12e). To address this energy deficit, the battery switches to discharge mode to compensate for the shortfall and ensure stable system operation (Fig. 12c). This phase underscores the crucial role of energy storage in maintaining a balance between generation and consumption, particularly in situations where renewable energy production decreases. Throughout these different phases, the *PV* voltage and the wind turbine’s rotational speed adapt and follow their optimal values to maximize energy extraction from the available renewable sources (Fig. 12b). This optimized regulation ensures a high system efficiency and effective management of energy flows.

### Case without energy generation

**Fig. 13:**
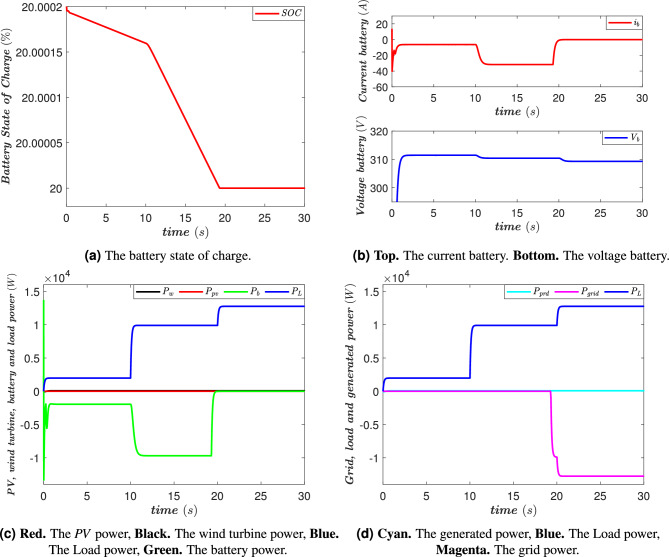
The power flow management system’s performance in the case without energy generation.

In this case, it is assumed that the energy production from the *PV* and the wind turbine is completely zero. This situation may be due to unfavorable weather conditions, such as insufficient sunlight or a lack of wind, preventing these renewable sources from generating electricity. During the time interval [0, 10 s], the battery plays a key role in supplying power to the *AC* loads. Since no energy is produced by the *PV* and the wind turbine, the battery becomes the sole energy source available to meet the system’s needs, as illustrated in Fig. 13a. During this period, the battery discharges gradually to provide the necessary power to the connected loads. Between [10, 20 s], the load consumption increases, leading to an intensification of the battery discharge process. This evolution is represented in Fig. 13c, where a rise in the power demand of the loads can be observed. The battery then adjusts its discharge current to meet this additional demand, as shown in Fig. 13a and b. This situation highlights the importance of energy storage in an isolated system, particularly when renewable sources are unavailable. At t = 19.4s, the battery’s state of charge reaches its minimum level, meaning it can no longer continue supplying energy. This critical condition forces the system to switch to an alternative power source to ensure service continuity. At this moment, the *AC* grid automatically takes over and begins supplying power to the loads, thus maintaining system stability and preventing any power interruption. This transition is clearly visible in Fig. 13c and d, where the power supplied by the grid increases to compensate for the battery?s depletion. Then, during the interval [20, 30 s], the entire power demand of the *AC* loads continues to be supplied by the *AC* grid. This phase illustrates the necessity of a backup power source in a microgrid, particularly when renewable sources are unavailable, and energy storage reaches its limits. The integration of the *AC* grid thus prevents any loss of power supply and ensures stable system operation, guaranteeing uninterrupted energy for the loads.

### Case of full battery

**Fig. 14:**
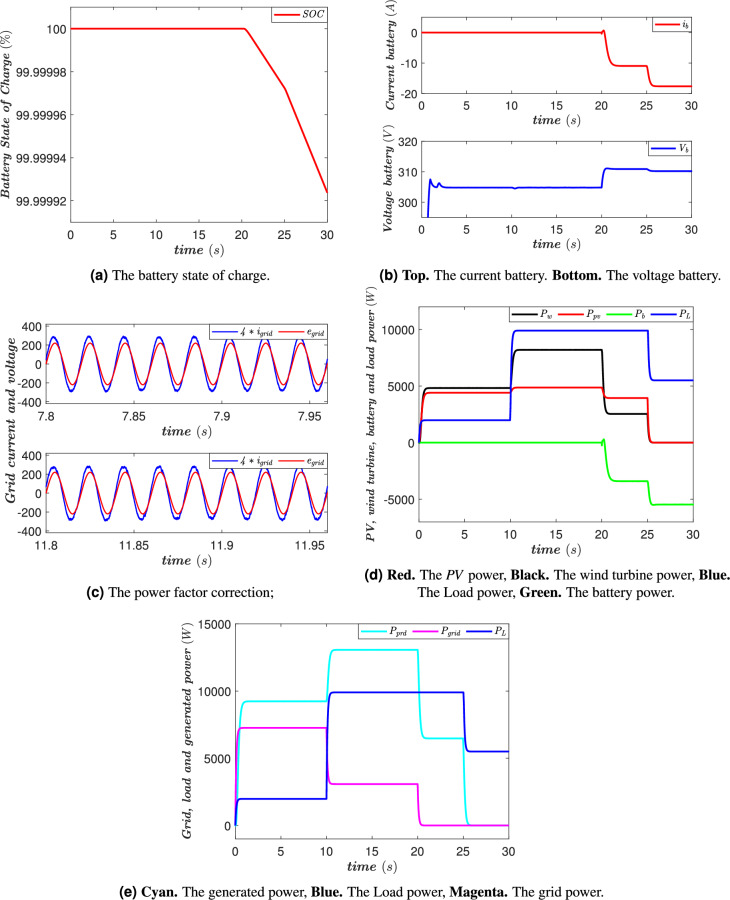
The power flow management system’s performance in the case of full battery.

In this case, the battery is fully charged, as indicated in Fig. 14a and b. During the time interval [0 10 s], the renewable energy sources, generate energy. This production is sufficient to meet the power demand of the *AC* loads. Moreover, the excess energy generated by these renewable sources is injected into the grid, as illustrated in Fig. 14e. This situation reflects an optimal operation of the system, where energy production exceeds consumption, allowing the surplus energy to be utilized efficiently. Between [10 20 s], a gradual increase in the consumption of *AC* loads is observed. However, this rise in power demand is entirely managed by the renewable energy sources. Indeed, despite the increase in consumption, energy production remains higher than the system’s needs. This means that the *PV* panel and the wind turbine not only continue to supply the *AC* loads but also inject the excess energy into the grid. This phase demonstrates the system’s ability to adapt to fluctuations in demand without requiring the intervention of the battery. During the time interval [20 25 s], the situation gradually changes. The energy produced by the *PV* panel and the wind turbine becomes lower than the demand of the *AC* loads, as illustrated in Fig. 14d. At this point, the system must compensate for this energy deficit to ensure a stable and reliable operation of the *AC* loads. In this context, the battery comes into action: it begins to discharge to supply the missing power, as shown in Fig. 14a and b. This operating mode ensures an uninterrupted power supply to the loads, thus guaranteeing energy continuity even in the event of a drop in renewable energy production. During the time interval [25 30 s], the *PV* panel and the wind turbine no longer produce power. This situation may be due to unfavorable weather conditions, such as insufficient sunlight or a lack of wind. In this case, the battery becomes the sole energy source to power the *AC* loads. It continues to discharge to meet the power demand, as indicated in Fig. 14a and b. Throughout these phases, the Power Factor Correction (*PFC*) is maintained on the *AC* grid side (Fig. 14c).

### Performance analysis and quantitative metrics

In this section, a quantitative analysis of system performance is described. Key metrics will be used to evaluate *energy efficiency*, *loss reduction* and *power factor improvement*. These metrics are calculated from the simulation results obtained in the scenarios studied (cases with energy production, without production, and full battery).

#### Energy efficiency

Energy efficiency ($\eta$) represents the ratio between the actual power used by the load and the total power generated by renewable sources. It is defined as follows:

40 $$\begin{aligned} \eta = \frac{P_{\text {load}}}{P_{\text {generated}}} \times 100 \end{aligned}$$

where $P_{\text {load}}$ is the power consumed by the loads and $P_{\text {generated}}$ is the total power produced by the system.

Equation (40) expresses the instantaneous power efficiency, relating input and output power at a given time step. For a rigorous assessment, the energy efficiency is defined as the ratio of the total output to input energy over the operating horizon. In this work, all reported efficiencies are evaluated over the complete simulation period, ensuring that the results reflect time-Equation (40) expresses the instantaneous power efficiency, relating input and output power at a given time step. For a rigorous assessment, the energy efficiency is defined as the ratio of the total output to input energy over the operating horizon.

$$\begin{aligned} \eta _E = \frac{\int _{t_0}^{t_f} P_{\text {out}}(t)\, dt}{\int _{t_0}^{t_f} P_{\text {in}}(t)\, dt}, \end{aligned}$$

where $P_{\text {out}}(t)$ and $P_{\text {in}}(t)$ are the instantaneous output and input powers, respectively, and $[t_0, t_f]$ is the considered operating horizon. In this work, all reported efficiencies are evaluated over the complete simulation period, ensuring that the results reflect time-dependent energy conversion performance.

Analysis of the simulation results shows that :

- In the case of sufficient energy production, **energy efficiency exceeds 90%**, showing a good match between supply and demand.
- When consumption exceeds available production, efficiency falls slightly (around **75%−80%**), indicating that energy from the grid or the battery compensates for the deficit.

#### Loss reduction

In the case where the grid is unavailable but renewable sources and storage are active, the power losses ($P_{\text {losses}}$) correspond solely to the excess energy that cannot be consumed or stored. They are defined as :

41 $$\begin{aligned} P_{losses} = P_{generated} - (P_{load} + P_{battery}) \end{aligned}$$

where $P_{\text {battery}}$ is the power stored (if positive) or restored (if negative) by the battery.

Analysis of the simulations shows that :

- Average losses are negligible ($-220 W$ i.e. less than 10%.), indicating efficient energy management.
- Maximum losses reach **4000** *W* when excess energy cannot be stored immediately.
- Minimum losses are **negative** ($-6000 W$), which means that demand sometimes exceeds production and the battery compensates for this deficit.

These results confirm that the system effectively optimises the use of available energy sources. However, in the absence of a network to absorb surpluses, some renewable energy remains unused in certain cases.

#### Power factor

The power factor (PF) is an indicator of the quality of the power supply. It is defined as :

42 $$\begin{aligned} PF = \frac{P_{\text {active}}}{S} \end{aligned}$$

where $P_{\text {active}}$ is the active power supplied to the load and *S* is the total apparent power.

The simulations show that:

- The power factor remains above **0.95** for most of the time, indicating a good match between active and reactive power.
- During transitions (e.g. battery $\Rightarrow$ grid), slight variations are observed but remain acceptable (greater than **0.9**).

#### Reliability quantification

In addition to stability proofs, the reliability of the proposed algorithm has been explicitly quantified through Key Performance Indicators (KPI) across four representative operating scenarios: with energy production, without production, full battery, and off-grid operation. Table 4 summarizes the results. These metrics confirm that the proposed strategy maintains high energy efficiency ($\ge$ 75%), low average losses (typically $< 15\%$), and a power factor consistently above 0.9, thereby ensuring robust and reliable operation under diverse operating conditions.

**Table 4:** Reliability quantification through KPI under different operating scenarios.

**KPI**	**With Energy Production**	**Without Energy Production**	**Full Battery**	**Off-Grid Case**	**Standard Reference**
Total Energy Produced (kWh)	High ($\sim$>150)	0	Moderate (>100)	Low (< 100, limited by storage and load constraints)	–
Energy Efficiency (%)	92	78	85	74	Compared with IEEE 1547 recommended efficiency benchmarks for DERs
Average Losses (%)	< 8	11	13	26.23	Acceptable levels consistent with IEC guidelines for system losses
Power Factor (PF)	0.97	0.93	0.96	0.91	IEEE 1547 requires PF$\ge$0.9 for grid-connected DERs

The performance analysis was benchmarked against international standards. Efficiency and power factor results comply with IEEE 1547 requirements for distributed energy resources (PF $\ge$ 0.9), while power loss evaluation follows IEC guidelines (IEC 61850, IEC 60364-4). Thus, the proposed strategy is validated both in simulations and against recognized international benchmarks for microgrid efficiency and reliability.

#### Summary of results

Table 5 summarises the average values of the performance metrics for the different scenarios tested:

**Table 5:** Summary of the system’s performance metrics.

**Scenario**	**Energy Efficiency (%)**	**Average Losses (%)**	**Power Factor (PF)**
With Energy Production	92%	< 8%	0.97
Without Energy Production	78%	11%	0.93
Full Battery	85%	13%	0.96
OFF Grid Case	74%	**26.23%**	0.91

These results confirm that the proposed control and energy management strategy ensures an **effective optimization of production and storage** while maintaining **a high quality of power supply**. The losses remain minimal in most scenarios, except when the grid is unavailable, where energy surpluses that cannot be stored lead to increased losses.

## Conclusion

In this study, we propose a nonlinear control approach coupled with an energy management algorithm for a hybrid system combining solar photovoltaic and wind energy, along with an energy storage device. This system consists of a photovoltaic (*PV*) generator, a wind turbine, and a lithium-ion battery, all successively connected to a single-phase grid through inverters, as well as to *AC* loads. First, the system dynamics are modeled using an averaged nonlinear state-space model, defined by equations (1a)-(1d), (2a)-(2d), and (3a)-(3c). Subsequently, a control strategy is developed to achieve several performance objectives: (i) Regulation of renewable energy source voltages: The voltages from the photovoltaic generator ($V_{pv}$) and the wind turbine ($V_{w}$) must precisely follow their respective optimal reference values ($V_{pvopt}$ and $V_{wopt}$). These reference values are dynamically computed by dedicated optimizers, ensuring that both systems operate at their maximum efficiency points. (ii) Power factor correction (*PFC*): The currents injected into the grid must be purely sinusoidal and in phase with the grid voltage, ensuring efficient operation and compliance with power quality standards. (iii) Constant Current (*CC*) Mode: During this stage, the battery current $i_b$ is regulated to follow a constant reference value $i^*_{b}$, which represents the maximum allowable charging current. This mode continues until the battery voltage reaches its maximum charging threshold, typically corresponding to about $70{-}80\%$ of the battery *SOC*. Constant Voltage (*CV*) Mode: Once the maximum battery voltage is reached, the control switches to this mode. At this stage, the voltage is maintained constant at a fixed reference value $V^*_{b}$, while the charging current gradually decreases. When the battery *SOC* reaches 100%, the charging current becomes nearly zero. To achieve these control objectives, a multi-loop nonlinear control strategy based on Backstepping is implemented. Furthermore, the reliability of the proposed control and energy management strategy has been explicitly quantified. Both theoretical stability analysis (Lyapunov proofs) and KPI-based evaluation confirm that the system maintains uninterrupted and robust operation under all considered scenarios, ensuring practical applicability. Finally, numerical simulations are performed in the MATLAB/SIMULINK/SIMPOWER environment to evaluate the performance of the proposed control scheme. The obtained results confirm that the control objectives are successfully met, demonstrating a satisfactory dynamic response and high system efficiency.

## Data Availability

The data that supports the findings of this study are available from the corresponding author on request.

## Abbreviations

$$V_{pv}$$: *PV* voltage
$V_{w}$: Wind turbine voltage
$V_{b}$: Battery terminal voltage
$$V_{g}$$: grid voltage
$U_{ocv}$: Battery open circuit voltage
$$i_{pv}$$: *PV* current
$i_{rec}$: Wind turbine current
$$i_{b}$$: Battery current
$$i_{L}$$: Loads current
$$i_{g}$$: Grid current
$R_{s}$: Battery internal resistance
$$R_{p}$$$$C_{p}$$: Battery long term transient polarization effect
*SOC*: State of Charge
*MPPT*: Maximum Power Point Tracking
*APPT*: Adaptive Power Point Tracking
*KPI*: Key Performance Indicator
*EFM*: Energy Flow Management
$$P_{L}$$: Loads power
$$P_{b}$$: Battery power
$$P_{prod}$$: Produced power
*LF*: Lyapunov function
*CC*: Constant Current
*ACC*: Adaptive Constant Current
*CV*: Constant Voltage
*PWM*: Pulse Width Modulation
$$\omega _{g}$$: Grid pulsation
*x*: Average state
$\mu$: Average values of D over cutting period PWM
*D*: Duty ratio function
